# Application of microfluidic chip-based multiplex PCR in diagnosing reproductive tract pathogens among patients with premature rupture of membranes

**DOI:** 10.3389/fcimb.2025.1722768

**Published:** 2026-01-06

**Authors:** Liuyan Zhang, Zhoujie Shao

**Affiliations:** 1 Department of Obstetrics, Beilun People’s Hospital, Ningbo, China; 2 Department of Clinical Laboratory, Beilun People’s Hospital, Ningbo, China

**Keywords:** premature rupture of membranes (PROM), microfluidic chip, multiplex PCR, reproductive tract pathogens, diagnostic performance

## Abstract

**Objective:**

To evaluate the diagnostic performance of a microfluidic chip-based multiplex PCR method for detecting common reproductive tract pathogens in patients with premature rupture of membranes (PROM).

**Methods:**

A total of 200 patients with PROM were recruited from January 2020 to December 2024. Clinical samples were collected and analyzed using a microfluidic chip-based multiplex PCR method to detect six common reproductive tract pathogens. Clinical etiological diagnosis was used as the reference standard to assess the diagnostic efficacy of the method. Sensitivity, specificity, positive predictive value (PPV), negative predictive value (NPV), and consistency were calculated using 2 × 2 contingency tables. Receiver operating characteristic (ROC) curve analysis was performed to further evaluate the diagnostic performance.

**Results:**

The mean age of the patients was 30.5 years ± 4.2 years, and the mean gestational age was 32.7 ± 3.4 weeks. The most frequently detected pathogen was *Escherichia coli* (28.0%), followed by *Candida albicans* (21.0%) and *Chlamydia trachomatis* (19.0%). The microfluidic chip-based multiplex PCR method demonstrated high sensitivity (95.6%), specificity (98.2%), PPV (94.2%), and NPV (97.8%). ROC curve analysis revealed excellent diagnostic performance for all pathogens, with area under the curve (AUC) values ranging from 0.93 to 0.98.

**Conclusion:**

The microfluidic chip-based multiplex PCR method provides a highly sensitive, specific, and reliable diagnostic tool for detecting reproductive tract pathogens in patients with PROM.

## 1 Introduction

Premature rupture of membranes (PROM) represents a significant complication within the field of obstetrics, with an incidence rate observed to be approximately 5% to 15% globally (Huang et al., 2018). It is characterized by the spontaneous rupture of the amniotic membranes before the onset of labor, often leading to preterm birth. PROM is associated with a range of adverse outcomes for both the mother and the neonate, including intra-amniotic infection, chorioamnionitis, and neonatal sepsis (Boskabadi et al., 2024) (Cao et al., 2023). The etiology of PROM is complex and multifactorial, yet reproductive tract infections have been identified as one of the most substantial risk factors (Lin et al., 2024). These infections can undermine the integrity of the fetal membranes. The underlying mechanism involves the initiation of inflammatory responses, which in turn activate metalloproteinases. The activation of these enzymes leads to collagen degradation and a consequent weakening of the membranes (Qian et al., 2023) (Bouvier et al., 2019). Given the far - reaching consequences of PROM, it is of paramount importance to achieve early and accurate diagnosis of these infections. Such timely and precise diagnosis serves as a cornerstone for effective management strategies and is crucial in driving improved clinical outcomes (Ronzoni et al., 2022).

Traditional diagnostic methods for reproductive tract infections, such as bacterial culture and serological tests, have limitations in terms of sensitivity, specificity, and turnaround time (Caruso et al., 2021). Molecular techniques, including polymerase chain reaction (PCR), have shown promise in overcoming these limitations by providing rapid, sensitive, and specific detection of pathogens (Batool and Galloway-Peña, 2023). However, the application of PCR in clinical practice is often restricted by the need for specialized equipment and technical expertise.

Recent advancements in microfluidic technology have led to the development of microfluidic chip-based platforms that offer a more accessible and efficient alternative for molecular diagnostics (Li et al., 2022). These platforms integrate sample processing, amplification, and detection in a single device, reducing the complexity and time required for PCR-based assays. Multiplex PCR, which allows the simultaneous detection of multiple pathogens in a single reaction, further enhances the diagnostic capabilities of these platforms (Zhang et al., 2025).

We designed a multiplex PCR panel that targets pathogens commonly linked to PROM and adverse obstetric outcomes, with varying culture recoveries. In this study, we aim to evaluate the diagnostic performance of a microfluidic chip-based multiplex PCR method for detecting common reproductive tract pathogens in patients with PROM. We hypothesize that this method will provide high sensitivity, specificity, and rapid turnaround times, making it a valuable tool for clinical diagnosis and management of PROM-related infections.

## 2 Method

### 2.1 Study subjects

Patients with PROM who met the inclusion criteria were selected as study subjects in our hospital from January 2020 to December 2024. The inclusion criteria were based on the diagnostic standards for PROM from the guidelines of the American College of Obstetricians and Gynecologists (ACOG) (2020) (Prelabor rupture of membranes: ACOG practice bulletin summary, number 217, 2020), and the patients were required to meet the following conditions: aged between 18 and 45 years, with a gestational age of at least 28 weeks, no history of antibiotic, antiviral drug, or antiseptic use (systemic or topical) within two weeks before admission, and signed informed consent. Assuming an expected sensitivity of 95% and an allowable margin of error of 5%, a minimum of 182 evaluable cases was required; to allow for exclusions, we planned to screen ≈ 220 participants. Target-species selection was based on published epidemiological data linking these six organisms to PROM, chorioamnionitis, or preterm delivery, and on their variable culturability.

The study protocol received approval from the Institutional Review Board of Beilun Branch Hospital of the First Affiliated Hospital of Medical School Zhejiang University (Approval No: KY20220243), and conducted in accordance with the Declaration of Helsinki. All participants provided written informed consent to participate in this research.

### 2.2 Sample collection

The six-target panel comprises *E. coli, C. albicans, C. trachomatis, GBS, M. hominis and S. pneumoniae*—the organisms that accounted for >95% of culture/NAAT-positive results in our previous two-year obstetric cohort and cover a range of culture recovery rates. For each study subject, two sterile cotton swab samples of cervical canal secretions were collected using a vaginal speculum. Two sterile flocked swabs were consecutively inserted into the cervical canal under direct visualization by a single examiner. To minimize sampling variability, neither the order of insertion (left vs. right) nor the sequence (first vs. second) was pre-assigned; the swab for culture and the swab for PCR were therefore drawn from the same anatomical region and were exposed to virtually identical secretions. Both swabs were rotated through 360°for 5 s and withdrawn immediately. One sample was used for bacterial and mycoplasma culture, as well as chlamydia detection, and the other was analyzed using a multiplex PCR assay based on microfluidic chip technology. Strict aseptic techniques were followed during sample collection to prevent cross-contamination.

### 2.3 Detection method

#### 2.3.1 Multiplex PCR amplification

Multiplex PCR amplification: A multiplex PCR technique with pathogen-specific primers was employed to simultaneously amplify six common reproductive-tract pathogens using hot-start Taq DNA polymerase (2.5 U per 25 μL). Primer sequences, concentrations and cross-reactivity validation are given in **Supplementary Table 1** and **Supplementary Figure 1**.

#### 2.3.2 Reaction system

Reaction system: The multiplex PCR reaction mixture (25 μL) contained 1× multiplex PCR buffer (Tris-HCl 20 mM, KCl 40 mM, (NH_42_SO_4_ 100 mM, MgCl_2_ 3.5 mM), dNTPs 0.2 mM each, hot-start Taq DNA polymerase 2.5 U, SYBR Green I 0.5×, and gene-specific primers.

#### 2.3.3 Microfluidic chip detection

The microfluidic chip contains eight reaction wells, each preloaded with lyophilized reagents and species-specific primers for the target pathogens. After sample loading, the chip was sealed with an adhesive film. A mixture of 6.4 μL of template nucleic acid and 18 μL of reaction mix was injected into the two sample inlets.

The loaded chip was placed into a prototype microfluidic PCR cycler that integrates thermal control and real-time fluorescence detection. Cycling conditions were: initial denaturation at 95°C for 3 min, followed by 35 cycles of 95°C for 5 s, 60°C for 20 s (with fluorescence acquisition), and 72°C for 15 s. Total run time was approximately 55 minutes. To prevent carry-over, chips were single-use with sealed inlet ports; no template was detectable in the subsequent blank run (n = 3 chip lots).

#### 2.3.4 Result interpretation

The accompanying software of the microfluidic chip detector automatically analyzed the results. The threshold line was typically set at 800. When the internal control showed an amplification curve with a Cq (quantification cycle) value of ≤ 32, the experimental results were considered valid. The positive control reaction wells and the internal control reaction wells exhibited typical S-shaped amplification curves, while the negative control reaction wells showed no amplification curves, and the internal control reaction wells displayed typical S-shaped amplification curves. If a project detection well showed a distinct amplification curve within 35 cycles and had a Cq value of ≤ 35, the corresponding detection project was judged as positive. If no distinct amplification curve appeared, it was judged as negative. The prototype chip reader is designed to integrate thermal cycling, fluorescence detection, and automated result interpretation, with the goal of reducing reliance on traditional laboratory equipment such as a qPCR machine or gel imager.

#### 2.3.5 Data analysis

The detection results were statistically analyzed to evaluate the sensitivity, specificity, and consistency of the multiplex PCR detection method. Clinical etiological diagnosis was used as the reference standard. 2 × 2 contingency tables and receiver operating characteristic (ROC) curves were used to evaluate the diagnostic efficacy.

### 2.4 Reference standard detection methods

The composite gold standard for each pathogen was defined as follows: *Escherichia coli*, *Streptococcus agalactiae* and *Streptococcus pneumoniae* were identified by aerobic culture on blood agar and MacConkey agar, followed by MALDI-TOF MS (Bruker Biotyper); *Candida albicans(conditional pathogen)* was isolated on Sabouraud dextrose agar and confirmed by microscopic morphology and CHROMagar Candida; *Chlamydia trachomatis* was detected using the FDA-approved Aptima Combo 2 NAAT (Hologic, USA, Ct ≤ 38); *Mycoplasma hominis* was cultured in Mycoplasma IST2 broth (bioMérieux, ≥10^4^ CCU/mL). All reference assays were performed by board-certified clinical microbiologists who were blinded to the chip results. When two reference methods for a given pathogen yielded discordant results (one positive and one negative), the sample was considered positive if either culture or pathogen-specific NAAT was positive (i.e., OR-rule); no tie-breaking weighting was applied. Discrepant outcomes were not sequenced due to resource constraints and are reported as-is to reflect real-world diagnostic accuracy. Because culture sensitivity differs among species, we additionally performed PCR-based detection for fastidious pathogens (*C. trachomatis*, *M. hominis*) and used these results as a complementary reference to reduce culture-related bias.

## 3 Result

### 3.1 Patient baseline characteristics

Among 218 women with premature rupture of membranes who were consecutively screened, 12 were excluded because of systemic or topical antibiotic/antiseptic use within the previous 14 days and 6 for inadequate specimen volume. Exactly 200 eligible participants remained, all of whom completed both the microfluidic-chip multiplex isothermal assay and the composite reference standard and were included in the final diagnostic-accuracy analysis (**Table 1**). The mean age of the patients was 30.5 years ± 4.2 years, and the mean gestational age was 32.7 ± 3.4 weeks. Participants had an average gravidity of 2.1 and parity of 1.0. A history of premature delivery was reported by 21.0% of the patients, and 34.0% had a history of genital tract infection.

**Table 1:** Baseline characteristics of patients.

Characteristic	Value
Age (years), mean ± SD	30.5 ± 4.2
Gestational age (weeks), mean ± SD	32.7 ± 3.4
Gravidity, mean ± SD	2.1 ± 1.3
Parity, mean ± SD	1.0 ± 0.9
History of premature delivery, n (%)	42 (21.0)
History of genital tract infection, n (%)	68 (34.0)

### 3.2 Pathogen detection rates

**Figure 1:**
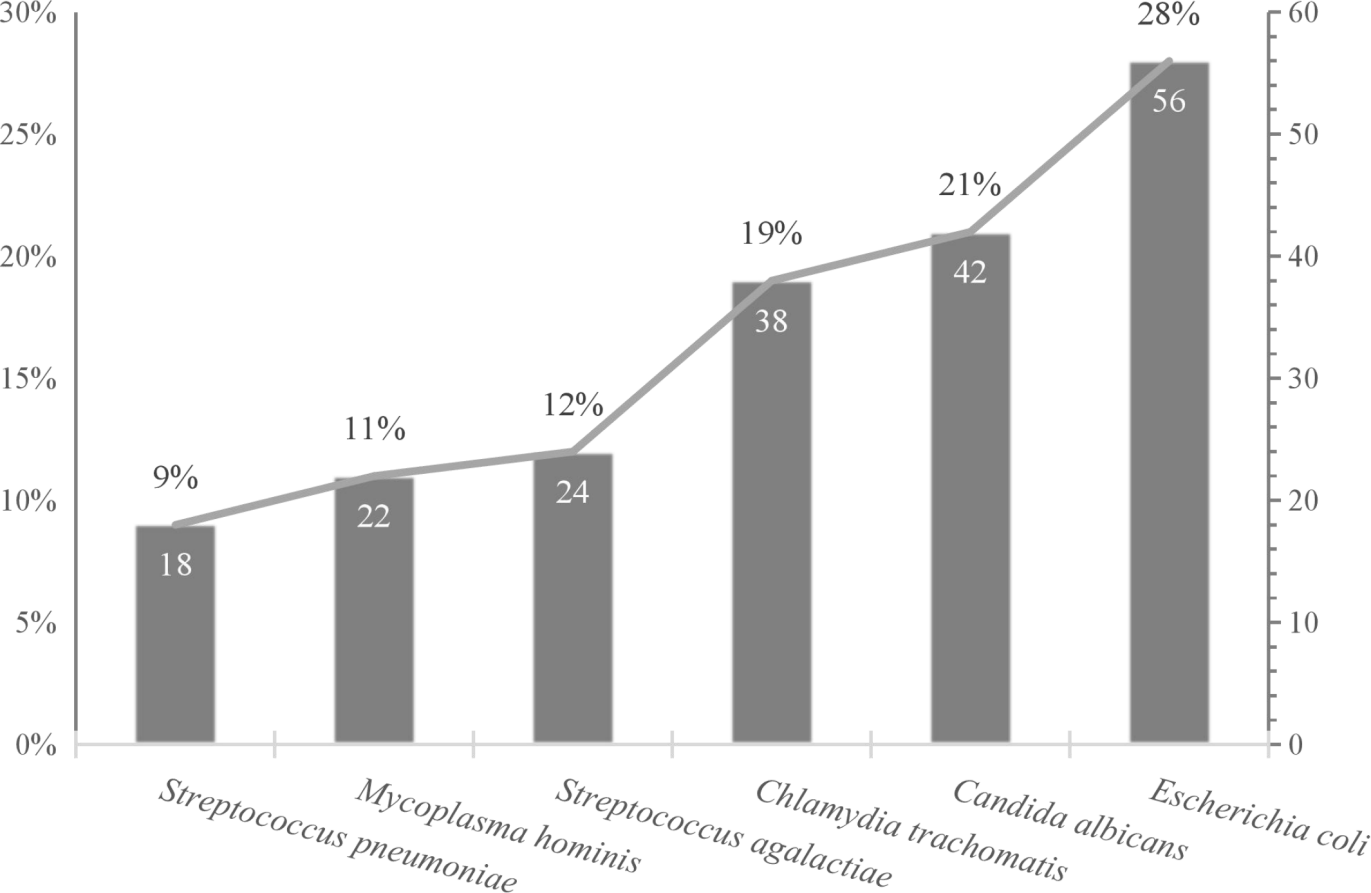
Pathogen distribution detected by multiplex PCR.

As shown in **Figure 1**, *Escherichia coli* was the most frequently detected pathogen, with a positive rate of 28%. This may reflect its significant role in causing reproductive tract infections and its potential association with PROM. *Candida albicans* and *Chlamydia trachomatis* also showed relatively high positive rates of 21% and 19%, respectively. The lower detection rates of *Streptococcus agalactiae* at 12%, *Mycoplasma hominis* at 11.0%, and *Streptococcus pneumoniae* at 9% indicate that while these pathogens are less common, they should not be overlooked in clinical practice (The observed prevalence of *M. hominis* (11%) and *C. trachomatis* (19%) should be interpreted as ‘PCR-recovery’ rather than true population prevalence, as culture sensitivity for these organisms is < 70%).

### 3.3 Diagnostic performance of the multiplex PCR

The sensitivity of 95.6% indicates that it can effectively identify most true positive cases of reproductive tract pathogen infections. The high specificity of 98.2% suggests a low rate of false positives, providing reliable negative results and minimizing unnecessary treatments or interventions. The positive predictive value (PPV) of 94.2% and negative predictive value (NPV) of 97.8% further confirm its robust performance in clinical practice.

**Figure 2:**
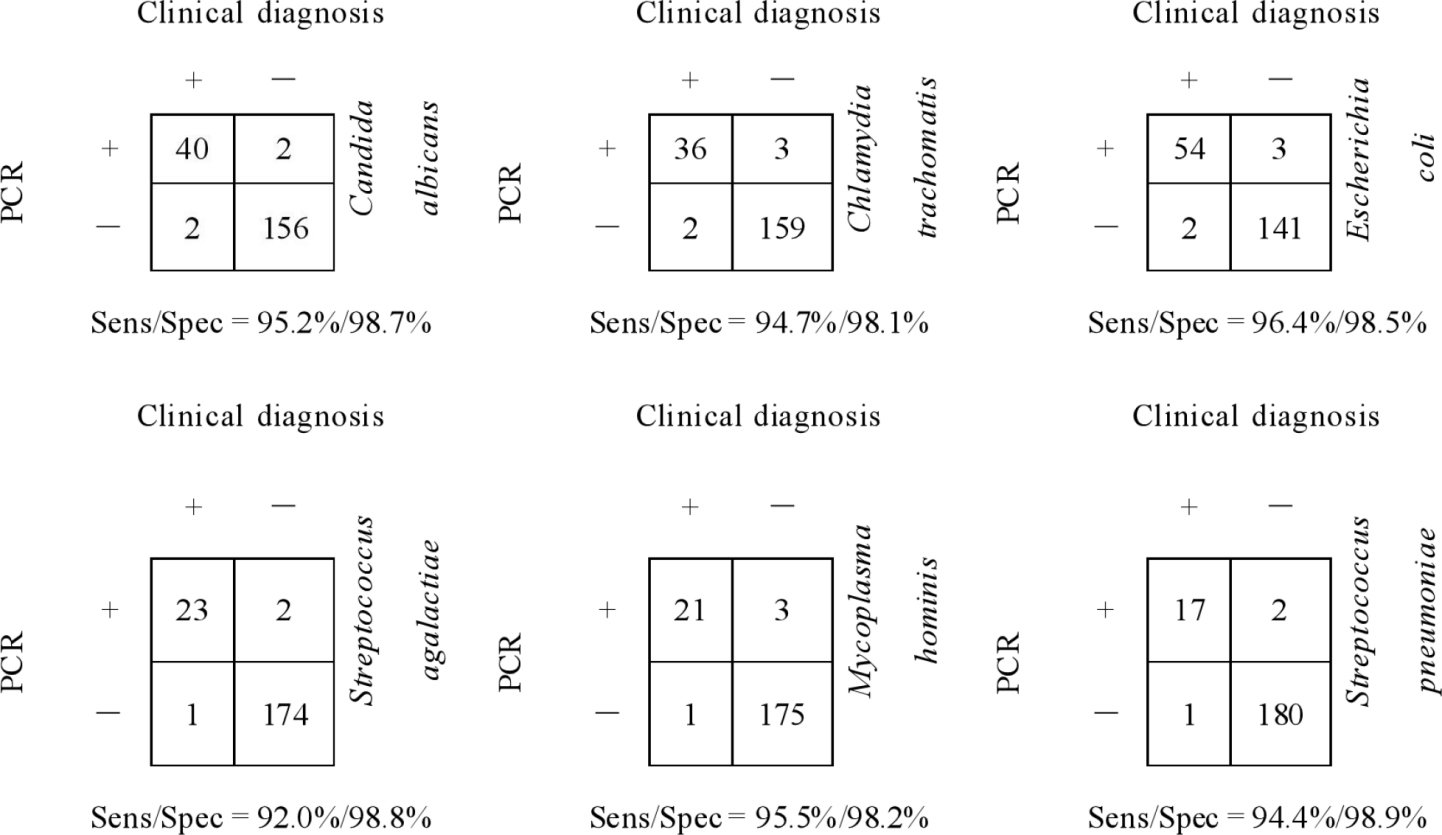
2 × 2 contingency table for diagnostic performance.

For *Candida albicans*, there were 40 true positive cases, 2 false positive cases, 2 false negative cases, and 156 true negative cases. The sensitivity was calculated as 95.2%, and the specificity as 98.7%. For *Chlamydia trachomatis*, the true positive count was 36, with 3 false positive, 2 false negative, and 159 true negative cases, yielding a sensitivity of 94.7% and specificity of 98.1%. In the case of *Escherichia coli*, 54 true positive cases, 3 false positive, 2 false negative, and 141 true negative cases were identified, resulting in a sensitivity of 96.4% and specificity of 98.5%. For *Streptococcus agalactiae*, the analysis revealed 23 true positive cases, 2 false positive, 1 false negative, and 174 true negative cases, with a sensitivity of 92.0% and specificity of 98.8%. *Mycoplasma hominis* showed 21 true positive cases, 3 false positive, 1 false negative, and 175 true negative cases, giving a sensitivity of 95.5% and specificity of 98.2%. Lastly, for *Streptococcus pneumoniae*, there were 17 true positive cases, 2 false positive, 1 false negative, and 180 true negative cases, leading to a sensitivity of 94.4% and specificity of 98.9%. Detailed two-by-two data are presented in **Figure 2**, and the corresponding pooled sensitivity, specificity and AUC with 95% CI are summarized in **Table 2**.

**Table 2:** Pooled diagnostic performance of the microfluidic-chip multiplex PCR for each target pathogen.

Pathogen	Sensitivity (95% CI)	Specificity (95% CI)	AUC (95% CI)
*Candida albicans*	95.2% (90.4–98.0)	98.7% (95.7–99.8)	0.96 (0.93–0.99)
*Chlamydia trachomatis*	94.7% (89.3–97.8)	98.1% (95.0–99.6)	0.94 (0.91–0.97)
*Escherichia coli*	96.4% (92.5–98.7)	98.5% (95.6–99.7)	0.98 (0.96–0.99)
*Streptococcus agalactiae*	92.0% (84.8–96.7)	98.8% (96.1–99.8)	0.95 (0.92–0.98)
*Mycoplasma hominis*	95.5% (88.5–98.7)	98.2% (95.3–99.6)	0.93 (0.89–0.97)
*Streptococcus pneumoniae*	94.4% (86.2–98.4)	98.9% (96.3–99.9)	0.97 (0.94–0.99)

CI, confidence interval; AUC, area under the ROC curve.

### 3.4 ROC curve analysis

ROC curve analysis further validated the diagnostic performance of the microfluidic chip-based multiplex PCR method. As summarized in **Table 3**, the area under the curve (AUC) values for all target pathogens ranged from 0.93 to 0.98, with Escherichia coli achieving the highest AUC of 0.98 (95% CI: 0.96–0.99). These results confirm that the method has excellent ability to distinguish between infected and non-infected patients, with minimal diagnostic uncertainty across all tested pathogens.

**Table 3:** LOD and AUC summary for target pathogens detected by microfluidic chip-based multiplex PCR.

Pathogen	Limits of detection (CFU mL^-^¹)	AUC (95% Confidence Interval)
*Escherichia coli*	1 × 10²	0.96 (0.93–0.99)
*Streptococcus agalactiae*	1 × 10²	0.94 (0.91–0.97)
*Candida albicans*	1 × 10²	0.98 (0.96–0.99)
*Chlamydia trachomatis*	1 × 10²	0.95 (0.92–0.98)
*Mycoplasma hominis*	1 × 10³	0.93 (0.89–0.97)
*Streptococcus pneumoniae*	1 × 10³	0.97 (0.94–0.99)

### 3.5 Analytical sensitivity (limit of detection, LOD)

To determine the LOD of the chip assay for each target pathogen, cervical-swab specimens previously found negative by both culture and chip were pooled and spiked with serial 10-fold dilutions (10¹–10^6^ CFU mL^-^¹) of freshly quantified reference strains. Each dilution was tested in triplicate on three independent days. The LOD was defined as the lowest concentration at which all three replicates were positive. LOD values in **Table 3** are reported as the nearest integer power of ten; individual end-point titrations ranged from 0.8 × 10² to 4.7 × 10³ CFU mL^-^¹ (**Supplementary Table 2**).

## 4 Discussion

The present study evaluated the diagnostic performance of a microfluidic chip-based multiplex PCR method for detecting common reproductive tract pathogens in patients with preterm PROM. Our results demonstrated that this method achieved high sensitivity (95.6%), specificity (98.2%), PPV (94.2%), and NPV (97.8%) when compared to clinical etiological diagnosis. These findings highlight the potential of the microfluidic chip-based multiplex PCR method as a reliable and efficient diagnostic tool for identifying reproductive tract infections in PROM patients.

Traditional diagnostic methods for reproductive tract infections, such as bacterial culture and serological tests, have been the cornerstone of clinical practice for many years. However, these methods have several limitations. Bacterial culture, for instance, can take several days to yield results, which delays the initiation of targeted antibiotic therapy (Santos et al., 2022). Serological tests, while faster, often lack the sensitivity and specificity required for accurate diagnosis, particularly in the context of PROM where early and precise intervention is crucial (Recommendations for the laboratory-based detection of Chlamydia trachomatis and Neisseria gonorrhoeae–2014, 2014).

In contrast, the microfluidic chip-based multiplex PCR method evaluated in this study provides rapid results, with the entire process from sample collection to result interpretation taking less than 2 hours. This is a significant improvement over traditional culture methods, which can take up to 48 hours or more (Bermudez et al., 2025). The high sensitivity and specificity of the microfluidic chip method (95.6% and 98.2%, respectively) are also superior to those of traditional methods. For example, studies have shown that traditional bacterial culture methods may have lower sensitivity and specificity for detecting certain pathogens. Contamination and other interfering factors can also affect the accuracy of the results (Wang et al., 2024).

*Escherichia coli* was the most frequently detected pathogen in this study, with a positive rate of 28.0%. This high prevalence is consistent with previous studies indicating that *E. coli* is a common cause of reproductive tract infections and is closely associated with PROM (Boutouchent et al., 2024). For instance, a study by Youssef et al. found that *E. coli* accounted for approximately 30% of all reproductive tract infections in pregnant women, highlighting its significant role in this context (Youssef et al., 2019). The microfluidic chip-based multiplex PCR method demonstrated excellent diagnostic performance for *E. coli*, with a sensitivity of 96.4% and specificity of 98.5%. This is particularly important given the potential complications associated with *E. coli* infections, such as intra-amniotic infection and chorioamnionitis, which can lead to preterm birth and neonatal sepsis (Shi et al., 2025). Early detection and treatment of *E. coli* infections using this method could significantly reduce the risk of these adverse outcomes.

In this study, *Candida albicans* was the second most commonly detected organism, with a positive rate of 21.0%. Candida infections can cause severe complications in both the mother and the neonate, including funisitis and neonatal candidiasis (Maki et al., 2017). The diagnostic performance of the microfluidic chip-based multiplex PCR method for *Candida albicans* was also impressive, with a sensitivity of 95.2% and specificity of 98.7%. Traditional diagnostic methods for Candida infections often rely on culture techniques, which can be time-consuming and have lower sensitivity. A study by Park et al. reported that culture methods for Candida detection had a sensitivity of only 60-80%, which is significantly lower than the sensitivity achieved by the microfluidic chip method (Park et al., 2025). This suggests that the microfluidic chip-based multiplex PCR method could be a more reliable tool for the early detection of Candida infections in PROM patients, enabling timely antifungal treatment and reducing the risk of complications.

*Chlamydia trachomatis* and *Mycoplasma hominis* were detected in 19% and 11% of the study participants, respectively. Both are linked to adverse pregnancy outcomes like PROM and preterm birth (Association of Chlamydia trachomatis and Mycoplasma hominis with intrauterine growth retardation and preterm delivery. The John Hopkins Study of Cervicitis and Adverse Pregnancy Outcome, 1989). Traditional detection methods (e.g., culture and DNA probes) have low sensitivity (only 60%–70% for *Mycoplasma*). In contrast, the microfluidic chip-based multiplex PCR method shows high sensitivity (95.5% for *Mycoplasma hominis* and 94.7% for *Chlamydia trachomatis*) and specificity (98.2% and 98.1%, respectively), making it a powerful tool for early intervention and improving pregnancy outcomes. This is particularly noteworthy as Chlamydia infections are often asymptomatic, making early detection challenging. A study by Johnson et al. highlighted that up to 70% of Chlamydia infections in pregnant women are asymptomatic, emphasizing the need for reliable diagnostic methods (Tang et al., 2019). The high sensitivity and specificity of the microfluidic chip method make it an ideal tool for identifying these silent infections, allowing for prompt treatment and reducing the risk of adverse pregnancy outcomes.

While PCR-based methods have been used for detecting reproductive tract pathogens, they often require complex laboratory setups and skilled technicians (Poritz and Lingenfelter, 2018). The microfluidic chip-based multiplex PCR method overcomes these challenges by integrating sample processing, amplification, and detection in a single, portable device. This integration not only reduces the risk of contamination but also simplifies the workflow, making it more accessible for routine clinical use (Li et al., 2025). Compared with single-plex qPCR, the microfluidic-chip multiplex PCR attains comparable analytical sensitivity (95.6%) while simultaneously detecting multiple pathogens in one reaction, reducing both sample volume and turnaround time. This is particularly beneficial in clinical settings where patients may be infected with more than one pathogen, as it provides a comprehensive overview of the infection status (Chen et al., 2023). Additionally, the microfluidic chip method requires smaller sample and reagent volumes, which can be cost-effective and reduce waste. Although the per-test cost of the microfluidic chip assay is slightly higher than that of culture methods (approximately ¥100–130 vs. ¥70–95), the reduction in labor, time, and potential clinical complications (e.g., delayed antibiotic therapy) makes it cost-effective in acute care settings. Mass production is expected to reduce chip costs to ¥30–50 per unit.

To better highlight our system’s novelty in PROM-specific diagnosis, we contrast it with recent microfluidic multiplex PCR/NAAT technologies. Li et al. ‘s microfluidic NAAT required 90 minutes of runtime and relied on laboratory-dependent equipment (Li et al., 2023); by contrast, our system shortens turnaround time to <60 minutes and uses a portable, battery-powered reader for labor ward deployment without central laboratory support. Zhang et al. reported an ultrafast (10-minute) multiplex PCR chip, but it focused on general infections (e.g., influenza) rather than PROM-specific pathogens and had a higher limit of detection (LOD: 1×10³ CFU/mL) than our assay (1×10² CFU/mL for *E. coli*, *Streptococcus agalactiae*, and *C. albicans*)—critical for detecting low-grade asymptomatic infections common in PROM (Zhang et al., 2025). Our LOD for fastidious pathogens like *Mycoplasma hominis* (1×10³ CFU/mL) also outperforms the 5×10³ CFU/mL reported by Chen et al. in their reproductive tract infection-focused platform, enhancing sensitivity for hard-to-culture organisms (Chen et al., 2023). Additionally, Bermudez et al.’s chip-based NAAT targeted urinary/bloodstream infections (not PROM) and required ≥10 μL of sample plus specialized preprocessing, whereas our assay uses only 6.4 μL of template and integrates lyophilized reagents to minimize hands-on time (Bermudez et al., 2025).

The microfluidic chip-based multiplex PCR system was designed for point-of-care testing scenarios. The chip reader is battery-powered and portable, requiring only a single sample-loading step. The total turnaround time from sample to result is <60 minutes, making it suitable for use in labor wards or outpatient clinics without the need for central laboratory infrastructure. The high diagnostic accuracy and rapid turnaround time of the microfluidic chip-based multiplex PCR method have significant clinical implications. Early and accurate diagnosis of reproductive tract infections can lead to timely and targeted antibiotic therapy, reducing the risk of adverse outcomes such as maternal sepsis, preterm birth, and neonatal complications (Daskalakis et al., 2023). The method’s ability to detect multiple pathogens simultaneously can also help in the management of polymicrobial infections, which are common in PROM patients (Dotters-Katz, 2020).

Future research should validate the microfluidic chip-based multiplex PCR in larger, more diverse populations to confirm generalizability. Although the number of discordant samples between the chip assay and the composite reference standard was small (n = 12, 6%), confirmatory sequencing could not be performed due to exhausted residual nucleic-acid extracts and limited resources; thus, misclassification bias cannot be fully excluded. The six-target panel reflects the predominant isolates recovered in our obstetric cohort; rarer pathogens were not included, and a negative result does not exclude these rarer pathogens. While we used a consecutive, non-ordered sampling protocol to minimize variability, the study design did not include a direct comparison of microbial yields between the two swabs, so subtle sampling effects cannot be ruled out. The composite reference remains culture-dependent for half of the targets; consequently, the prevalence of fastidious organisms such as *M. hominis* and *C. trachomatis* may be underestimated and their PCR-derived sensitivity potentially overestimated. Additionally, the absence of non-PROM controls limits prevalence comparisons and relative-risk estimates; future case–control studies are needed to quantify the true epidemiological burden of these pathogens. Although each run contained internal and positive controls that gave consistent Cq values (CV < 3%), formal intra-/inter-assay and inter-operator variability studies were not undertaken; multicentre evaluations will be needed to establish full reproducibility. Finally, this study evaluated diagnostic accuracy only; whether the chip-guided management improves antibiotic appropriateness, neonatal infection rates or other clinical endpoints awaits prospective interventional trials.

## 5 Conclusion

In conclusion, the microfluidic chip-based multiplex PCR method evaluated in this study showed excellent diagnostic performance for detecting reproductive tract pathogens in PROM patients. Its high sensitivity, specificity, and multiplexing capability make it a promising tool for rapid and accurate diagnosis of infections. Further research is needed to explore the full potential of this technology in clinical practice and to address the challenges associated with its widespread adoption.

## Data Availability

The original contributions presented in the study are included in the article/**Supplementary Material**. Further inquiries can be directed to the corresponding author.
