# Supplementary material for: Application of microfluidic chip-based multiplex PCR in diagnosing reproductive tract pathogens among patients with premature rupture of membranes
Source: Front Cell Infect Microbiol. 2026 Jan 6;15:1722768. doi: 10.3389/fcimb.2025.1722768 (PMC12816308; doi:10.3389/fcimb.2025.1722768)
Supplement: Supplementary file 1 [file Table1.docx]

### **Supplementary Table 1** LAMP primer sequences and final reaction concentrations

| **Pathogen** | **Target gene (accession)** | **Primer name** | **Sequence (5’→3’)** | **Stock (µM)** | **Volume (µL)** | **Final conc. in 25 µL (µM)** |
| --- | --- | --- | --- | --- | --- | --- |
| ****C. albicans**** | U46158 | CaR-F3-6 | TTATTAGCACTTCGTGAACAA | 90 | 0.5 | 1.8 |
|  |  | CaR-B3-6 | TGATTTGTCTGACAACATCAA | 90 | 0.5 | 1.8 |
|  |  | CaR-FIP-6 | CGGTCATCTTCTAAATCACACTTGT-GAGAATAAAAGATAGTGACAATGTC | 180 | 2.0 | 14.4 |
|  |  | CaR-BIP-6 | GGTGAAAGTGAGTCAAGATTGGG-TGAACGCTTCATCCACAT | 180 | 2.0 | 14.4 |
|  |  | CaR-LF-6 | TCCCAACCAATACCATAGG | 180 | 1.0 | 7.2 |
|  |  | CaR-LB-6 | CAAGTGCCATGTACAAAACA | 180 | 1.0 | 7.2 |
| ****C. trachomatis**** | HE603228 | CT-F3-1 | AAAGACAAAAAAGATCCTCGA | 90 | 0.5 | 1.8 |
|  |  | CT-B3-1 | GATTTCATGGGTAAAGGGATT | 90 | 0.5 | 1.8 |
|  |  | CT-FIP-1 | TGAAAAGCTTCTCCTTATTCGAAGTATGATCTACAAGTATGTTTGTTGAG | 180 | 2.0 | 14.4 |
|  |  | CT-BIP-1 | TGCGTTTCCAATAGGATTCTTGGGCAGCAAGAAATGTCGTTAG | 180 | 2.0 | 14.4 |
|  |  | CT-LF-1 | ATTATGCATTGGACCGCATCA | 180 | 1.0 | 7.2 |
| ****E. coli**** | CP054942 | Eco-F3-1 | GCTTTATGACTTGTAATAATGATGG | 90 | 0.5 | 1.8 |
|  |  | Eco-B3-1 | GGAAGAAATTCAACAAAACAACAG | 90 | 0.5 | 1.8 |
|  |  | Eco-FIP-1 | AAGGGGCCATTATGATCACTCACATTAACACCCTCTACAGAGAG | 180 | 2.0 | 14.4 |
|  |  | Eco-BIP-1 | AATCGTTCTTTCTTATCATCCACCAAACGACAGAAATTAATGCCAAC | 180 | 2.0 | 14.4 |
|  |  | Eco-LF-1 | GGAGATGCAAAAACTAAAGGAGGTA | 180 | 1.0 | 7.2 |
|  |  | Eco-LB-1 | TCTGTTTTAATGCATCTTCAGACGG | 180 | 1.0 | 7.2 |
| ****GBS**** | CP019814 | GBS-F3-2 | ATCAAGCCCAGCAAATGG | 90 | 0.5 | 1.8 |
|  |  | GBS-B3-2 | TTGCTTCAATCACATCTGTT | 90 | 0.5 | 1.8 |
|  |  | GBS-FIP-2 | CGGTTTTTCATAATCTGTTCCCTGACTCAAAAGCTTGATCAAGATAGC | 180 | 2.0 | 14.4 |
|  |  | GBS-BIP-2 | TAAAGACTTCATTGCGTGCCAGCTTCTACACGACTACCAAT | 180 | 2.0 | 14.4 |
|  |  | GBS-LB-2 | ACCCTGAGACAGTTTATGAT | 180 | 1.0 | 7.2 |
| ****M. hominis**** | CP055150 | MH-F3-3 | CACCCAATTTAAAATAATTGCTGATC | 90 | 0.5 | 1.8 |
|  |  | MH-B3-3 | GCAAAAAGATTTTCTTCATCCAA | 90 | 0.5 | 1.8 |
|  |  | MH-FIP-3 | GCCCTATATGCTCTTCTTATTAAACGTAGTTAATGCAATAAGTGACGG | 180 | 2.0 | 14.4 |
|  |  | MH-BIP-3 | TCTCTTGGCATAACTCAAAAAGCATTTTTCTTCAACATCAATGTCATAGA | 180 | 2.0 | 14.4 |
|  |  | MH-LF-3 | CCACGACCAATTTGAAGGTT | 180 | 1.0 | 7.2 |
| ****S. pneumoniae**** | MK606437 | SP-F3-7 | TTGTCAAAATAAGCGCCTAG | 90 | 0.5 | 1.8 |
|  |  | SP-B3-7 | ACTTCCTGGAAGACCCTAA | 90 | 0.5 | 1.8 |
|  |  | SP-FIP-7 | GCCTGGTTTGGAGTAGAAACCT-ATATTGCCAGAAGCATCATCA | 180 | 2.0 | 14.4 |
|  |  | SP-BIP-7 | ATGAGGGCTGGCATCCATTT-AAAGGTACATCCTAGTAGACC | 180 | 2.0 | 14.4 |
|  |  | SP-LF-7 | CTAACTTACACCTAGCCAT | 180 | 1.0 | 7.2 |
|  |  | SP-LB-7 | GGATGAGTTCTCCAGCA |  |  |  |

Final reaction volume = 25 µL. Concentrations: FIP/BIP 14.4 µM, F3/B3 1.8 µM, LF/LB 7.2 µM.

****Supplementary Figure 1**** the results of prime-specific amplification. Each primer set was challenged with non-target pathogens (10⁴ copies/µL); no non-specific amplification was observed.
