# Supplementary material for: Application of microfluidic chip-based multiplex PCR in diagnosing reproductive tract pathogens among patients with premature rupture of membranes
Source: Front Cell Infect Microbiol. 2026 Jan 6;15:1722768. doi: 10.3389/fcimb.2025.1722768 (PMC12816308; doi:10.3389/fcimb.2025.1722768)
Supplement: Supplementary file 2 [file Table2.docx]

**Supplementary Table 2**. Exact end-point titrations for LOD determination

| **Pathogen** | **Lowest 100 % positive titre (CFU mL⁻¹)** |
| --- | --- |
| Escherichia coli | 1.3 × 10² |
| Streptococcus agalactiae | 0.8 × 10² |
| Candida albicans | 2.1 × 10² |
| Chlamydia trachomatis | 0.9 × 10² |
| Mycoplasma hominis | 4.7 × 10³ |
| Streptococcus pneumoniae | 3.2 × 10³ |
